# Studying a Heterogeneous Array of Target Groups Can Help Us Understand Prejudice

**DOI:** 10.1177/0963721419830382

**Published:** 2019-04-02

**Authors:** Mark J. Brandt, Jarret T. Crawford

**Affiliations:** 1 Department of Social Psychology, Tilburg University; 2 Psychology Department, The College of New Jersey

**Keywords:** ideology, individual differences, personality, prejudice, representative stimuli

## Abstract

Prejudice can be expressed toward a wide array of target groups, but it is often operationalized as being expressed toward a narrower array of groups. By studying a heterogeneous array of target groups, we can draw broader conclusions about prejudice writ large. Here, we describe our research, in which we seek to understand constructs that consistently predict prejudice across a wide array of groups (consistent predictors), as well as constructs that predict prejudice for only some types of groups (inconsistent predictors). For inconsistent predictors, we can also identify the perceived characteristics of the target groups (e.g., status, ideology) that are associated with expressed prejudice. Studying a heterogeneous array of target groups opens up new questions related to morality, cognitive processing, and perceived discrimination but also suggests that prejudice, depending on the group, can be a motivating force preserving the status quo or prompting social change.

In this article, we review research on individual differences (e.g., political ideology, personality) and prejudice to illustrate how scholars can advance the study of prejudice and discrimination by studying a heterogeneous array of target groups. First, such research can help identify constructs that consistently predict prejudice across a wide array of groups (consistent predictors). Second, it can help identify constructs that predict prejudice for only some types of groups (inconsistent predictors). Third, for inconsistent predictors of prejudice, it can help identify the perceived characteristics of the target groups (e.g., status, ideology) that are associated with expressed prejudice.

## The Typical Prejudice-Assessment Strategy

The typical strategy in prejudice research is to measure or manipulate a particular construct, such as resource scarcity (Krosch, Tyler, & Amodio, 2017), violent video games (Greitemeyer, 2014), or impending doom (Quirin, Bode, Luckey, Pyszczynski, & Kuhl, 2014), and measure prejudice toward a particular group. This strategy has led to a number of findings: People express more prejudice when resources are scarce than abundant, after playing Call of Duty 2 (a war game) compared with Flipper (a pinball game), and when doom is impending than when it is not. Research examining generalized prejudice—the personality trait whereby people express more prejudice toward a variety of groups—uses more target groups (e.g., McFarland, 2010), yet these groups share a key feature: They are typically disadvantaged (Bergh, Akrami, Sidanius, & Sibley, 2016).

When studying prejudice, researchers often limit themselves to studying just a few different target groups and just a few different types of target groups. This is a problem. Prejudice can be expressed toward a large variety of target groups. Social psychologists define prejudice as a negative evaluation of a group or an individual based on group membership (e.g., Crandall, Eshleman, & O’Brien, 2002). This definition is not limited to specific subcategories of groups and applies to any possible group (e.g., African Americans, but also nerds). Although prejudice toward vulnerable groups may be the most consequential and vile (in our opinion), it is not the totality of prejudice. The well-accepted definition of prejudice we use focuses us on the core psychological issue: negative evaluations of a group.

If prejudice can be expressed toward any group, then research that focuses on a limited range of groups may provide misleading conclusions about prejudice. A hypothetical researcher might claim the threat of social upheaval increases prejudice but then measure prejudice only toward Arab Muslims. The finding may be preregistered, replicable, and robust according to all of the new norms of solid science (Munafò et al., 2017), but it cannot tell us about prejudice broadly. The same threat might decrease prejudice toward Whites, rich people, and people in the military and not affect prejudice toward Latinx or Filipino Americans. It is also possible that the threat does not increase prejudice toward Arab Muslims as predicted but does increase prejudice toward African Americans and gay men. If we instead include measures of prejudice toward a range of target groups, we can know whether the effect generalizes to other groups (increased prejudice), does not generalize to other groups (null effects), changes directions entirely (decreased prejudice), or emerges only with other groups (increased prejudice only for other groups). To make conclusions about the nature of prejudice broadly, beyond prejudice toward any specific group, researchers need to study prejudice as it is expressed toward a large number of groups.

## The Solution

There are options for increasing the heterogeneity of groups. We could study prejudice toward all possible social groups, from cheerleaders, rich people, and funeral home directors to African Americans, transgender people, and homeless people. The obvious challenge is that the number of social groups may approach infinity. A more manageable option is to include the range of target groups that the researchers hope will capture the necessary contours of the effects; those groups that are likely to show the hypothesized effect as well as those that might be less likely to show the effect (e.g., Craig & Richeson, 2014; Wetherell, Brandt, & Reyna, 2013). This can work, but it is easy to miss groups that may be relevant to individual participants. To address these shortcomings, we can use stimuli (target groups) representative of the population of interest (e.g., social groups in America; social groups at my university) and model these stimuli as random factors (Judd, Westfall, & Kenny, 2012). This ensures that results are not due to the particular characteristics of the groups included. And it ensures that we capture the psychological processes relevant to groups in people’s typical environment.

**Fig. 1.**
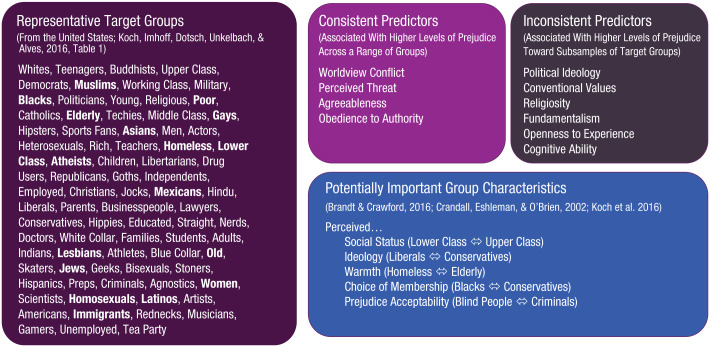
Representative target groups, consistent predictors of prejudice, inconsistent predictors of prejudice, and potentially important group characteristics. Representative target groups in the United States were generated by Koch, Imhoff, Dotsch, Unkelbach, and Alves (2016, Table 1). Boldface indicates groups that we think are more often studied in social-psychology research. Consistent predictors are associated with higher levels of prejudice across a range of groups. Inconsistent predictors are associated with higher levels of prejudice toward subsamples of target groups. Potentially important group characteristics are perceived characteristics of target groups that can be used to help understand when and why some inconsistent predictors are associated with prejudice instead of tolerance (Brandt & Crawford, 2016; Crandall, Eshleman, & O’Brien, 2002; Koch, Imhoff, Dotsch, Unkelbach, & Alves, 2016). The examples in parentheses are prototypical groups near the ends of each of the group-characteristic continua.

The benefits of representative stimuli are known (Brunswik, 1956; Wells & Windschitl, 1999) but have been applied only recently to the study of social groups.^1^ In particular, Koch, Imhoff, Dotsch, Unkelbach, and Alves (2016) developed techniques to identify representative samples of well-known social groups. In the typical case, participants generate a list of social groups in their country, which are used as stimuli in the main study. The task is purposefully ambiguous, without any group primes or examples, resulting in a list of groups that are commonly studied (e.g., Blacks, gays) but also some that are not commonly studied (e.g., athletes, nerds, hipsters; see Fig. 1) by psychologists (see Koch et al., 2016, for details). Other methods could identify groups important in other domains, such as intimacy groups (e.g., family, friends), task groups (e.g., coworkers), or other groups relevant in day-to-day life (Lickel et al., 2000).^2^

## The Findings

In our research, we use heterogeneous and representative samples of groups to understand predictors of prejudice. For organizational purposes, we chunk these predictors into constructs that consistently predict prejudice across a wide array of groups (consistent predictors) and constructs that predict prejudice for only some types of groups (inconsistent predictors).

### Consistent predictors of prejudice

**Fig. 2.**
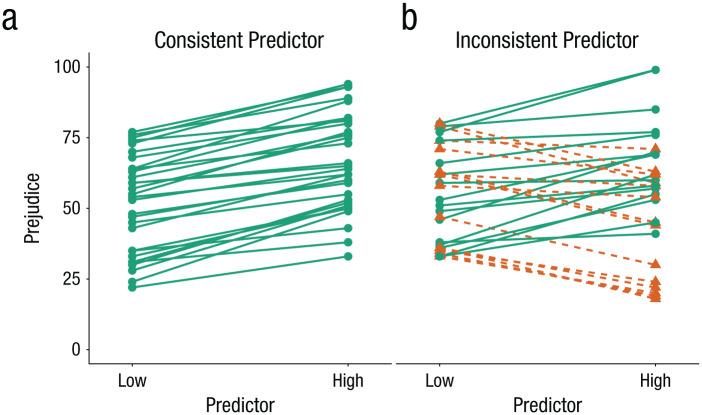
Association between a hypothetical predictor (at low and high levels) and prejudice toward a hypothetical target group in a single hypothetical study. Solid green lines show positive associations, and dashed orange lines show negative associations. Each of the 30 lines represents the association for a single group. Consistent predictors of prejudice (a) are associated with higher levels of prejudice across many target groups. Although the exact size of the relationship might differ, the effects all tend to be positive. Inconsistent predictors of prejudice (b) are associated with higher levels of prejudice for some target groups and lower levels of prejudice for other target groups. Sizes of these relationships will also vary. Perceived target-group characteristics can be used to explain the variation in the size and direction of these associations.

We have found evidence for at least four consistent predictors of prejudice; that is, characteristics of the target or perceiver that seem to predict prejudice consistently toward a variety of groups (Figs. 1 and 2a). The first consistent predictor we identified is *worldview conflict*, which is typically measured by asking people how much they see the targets as holding beliefs or values different from their own (e.g., Brandt, Chambers, Crawford, Wetherell, & Reyna, 2015; Crawford, Brandt, Inbar, Chambers, & Motyl, 2017; Wetherell et al., 2013). These perceptions are strongly associated with prejudice toward a wide range of target groups (Brandt et al., 2015; Crawford et al., 2017; Voelkel, Brandt, & Colombo, 2018). This effect is so consistent that it holds for people both high and low in Openness to Experience (Brandt et al., 2015), and puncturing the illusion of explanatory depth about people’s own worldviews does not reduce it (Voelkel et al., 2018).

Perceived threat—in terms of safety or resource competition—from the target is another consistent predictor of prejudice. Some perspectives (e.g., Jost, Stern, Rule, & Sterling, 2017) suggest that conservatism is especially tied to threat perceptions. However, our findings show that perceived threat from a group predicts prejudice among liberals and conservatives, as well as among religious fundamentalists and nonfundamentalists, when studies use a variety of prejudice measures (e.g., feeling thermometers, social-distance ratings, political intolerance; Brandt & Van Tongeren, 2017; Crawford, 2014). That said, there are sometimes ideological differences in the potency of different types of threats. For example, Crawford (2014) found that liberals’ intolerance is driven more by perceived threats to rights, whereas conservatives’ intolerance is driven more by threats to physical safety.

Worldview conflict and threat are perceivers’ perceptions of the target and so combine information about the target with the perceiver’s own perceptions and biases. There appear to be at least two additional consistent predictors of prejudice that are inherent to the perceiver. First, low scores on the Big Five trait Agreeableness predict prejudice against an assortment of groups, even after analyses control for other Big Five traits (Crawford & Brandt, in press), perhaps because people low in Agreeableness are less attuned to prejudice-suppressing norms (Graziano, Bruce, Sheese, & Tobin, 2007). Initial findings suggest that this is not an effect of overall negativity; low Agreeableness was not associated with negative evaluations of nonhumans (e.g., robots, frogs). Second, traits associated with obedience to authority predict political intolerance (but not prejudice per se) toward a range of activist groups on both the political left and right (Crawford, Mallinas, & Furman, 2015).

Notably, the findings for both of these traits push against conventional wisdom in the field. Whereas existing work shows that low Agreeableness is associated with prejudice against low-status groups (e.g., Sibley & Duckitt, 2008), our work using representative groups shows that this extends to high-status groups. Whereas existing work shows that obedience to authority predicts prejudice toward low-status and liberal groups (e.g., Altemeyer, 1998), our work using a variety of activist groups shows that this is also pernicious for high-status and conservative activist groups. These investigations are recent, and the question of what other (if any) traits or target characteristics predict prejudice against heterogeneous target groups remains low-hanging fruit for future research.

### Inconsistent predictors of prejudice

Although some factors (such as those described above) are associated with prejudice toward a range of groups, many factors are associated with prejudice toward targets groups that have only particular characteristics (Figs. 1 and 2b). They are not associated with prejudice in general and are instead associated with prejudice toward specific types of groups (e.g., liberals, conservatives, high-status groups). For these inconsistent predictors of prejudice, characteristics of the target group may turn off or even reverse the relationship between the predictor and prejudice (Fig. 1).

The example we have studied most often is the association between political ideology (sometimes called ideological identification) and prejudice. Prior work suggests that political conservatives and people with more traditional worldviews express more prejudice than liberals and people with more progressive worldviews (e.g., Sibley & Duckitt, 2008). However, using heterogeneous target groups, we found that the relationship between conservatism and prejudice reversed depending on the perceived ideology of the target group (see Brandt, Reyna, Chambers, Crawford, & Wetherell, 2014, for an initial review). These findings have been extended to different dimensions of political ideology (i.e., social and economic; Crawford et al., 2017; Czarnek, Szwed, & Kossowska, 2018), ideological worldviews (i.e., right-wing authoritarianism and social-dominance orientation; Crawford et al., 2015), and religious fundamentalism (Brandt & Van Tongeren, 2017; Kossowska, Czernatowicz-Kukuczka, & Sekerdej, 2017) and held when using representative target groups (Brandt, 2017). In each case, people on the political left express prejudice toward people perceived to be on the political right, and people on the political right express prejudice toward people perceived to be on the political left. This is because people experience worldview conflict and various threats from ideological out-groups. And these results hold when analyses controlled for other group characteristics, such as perceived social status or choice of being a member of the group (Brandt, 2017).^3^

Existing prejudice models did not anticipate that political liberals and conservatives both express similar levels of prejudice toward different groups. This is because low Openness and cognitive ability are associated with prejudice, and political liberals report being more open to experiences and have higher levels of cognitive ability than political conservatives (e.g., Onraet et al., 2015; Sibley & Duckitt, 2008). However, we find that Openness to Experience and cognitive ability do not make one immune: Openness and cognitive ability are both associated with prejudice against socially conventional groups (Brandt et al., 2015; Brandt & Crawford, 2016). People with low levels of cognitive ability also tend to express more prejudice against groups in which group membership is not perceived to be the group member’s choice (e.g., ethnic groups, as opposed to religious groups; Brandt & Crawford, 2016). None of these findings suggest that previous research was incorrect but rather that it was incomplete. When more groups are included, a more complete picture emerges.

## Extensions and Future Directions

Heterogeneous target groups also help us investigate other research questions and domains. Using a heterogeneous array of groups has elucidated (a) how political extremism is associated with prejudice and negative emotions (van Prooijen, Krouwel, Boiten, & Eendebak, 2015), (b) when liberals or conservatives are likely to respect authority (Frimer, Gaucher, & Schaefer, 2014), and (c) the extent to which partisans categorize political reality into simpler and homogenous categories (Lammers, Koch, Conway, & Brandt, 2017). One possible area of inquiry is the negative consequences of perceived prejudice on well-being for people from a variety of groups (e.g., Pascoe & Smart Richman, 2009). It may be that some groups (e.g., high-status groups) are less affected by perceived prejudice because of the other social and financial resources they can draw on. Such findings would challenge narratives and beliefs of majority-group victimization (cf. Norton & Sommers, 2011).

Prejudice is typically associated with preserving the status quo and maintaining intergroup inequality (e.g., support for racist and sexist policies). Studying prejudice toward a heterogeneous sample of groups highlights that prejudices toward some groups could also serve as motivation for social change. Just as prejudice toward low-status groups discourages support for policies redressing inequality, prejudice toward high-status groups may inspire support for economically redistributive or reparative social-justice policies. Although a politics underpinned by prejudices may be corrosive overall, using heterogeneous target groups makes it possible to understand prejudice as both an agent of support for the status quo and an agent for social change.

## Recommended Reading

Brandt, M. J. (2017). (See References). A series of studies in which representative samples of groups were used to develop and test a predictive model of ideological prejudice.

Crawford, J. T., Brandt, M. J., Inbar, Y., Chambers, J. R., & Motyl, M. (2017). (See References). A series of studies in which heterogeneous samples of groups were used to identify differences and similarities in how economic and social political ideologies are associated with prejudice.

Fiedler, K. (2011). Voodoo correlations are everywhere—Not only in neuroscience. *Perspectives on Psychological Science*, 6, 163–171. A commentary highlighting how experimental-design choices, including the choice of stimuli, can inflate effect sizes and bias results.

Koch, A., Imhoff, R., Dotsch, R., Unkelbach, C., & Alves, H. (2016). (See References). A series of studies in which representative samples of groups were used to map consensus group stereotypes.

## Notes

1. There are calls for representative stimuli in political psychology more broadly (Baron & Jost, 2019; Brandt & Wagemans, 2017; Kessler, Proch, Hechler, & Nägler, 2015).

2. We thank two anonymous reviewers for these suggestions.

3. When it comes to empathy, rather than prejudice, status may play more of a role (Lucas & Kteily, 2018).
